# External respiratory motion analysis and statistics for patients and volunteers

**DOI:** 10.1120/jacmp.v14i2.4051

**Published:** 2013-03-04

**Authors:** Sarah Quirk, Nathan Becker, WL Smith

**Affiliations:** ^1^ Department of Medical Physics Tom Baker Cancer Centre Calgary AB; ^2^ Department of Physics and Astronomy University of Calgary Calgary AB; ^3^ Department of Radiation Physics Princess Margaret Hospital Toronto ON; ^4^ Department of Radiation Oncology University of Toronto Toronto ON; ^5^ Department of Oncology University of Calgary Calgary AB Canada

**Keywords:** respiratory motion, inter‐ and intrafraction motion, variability, baseline drift

## Abstract

We analyzed a large patient and volunteer study of external respiratory motion in order to develop a population database of respiratory information. We analyzed 120 lung, liver, and abdominal patients and 25 volunteers without lung disease to determine the extent of motion using the Varian Real‐Time Position Management system. The volunteer respiratory motion was measured for both abdominal and thoracic placement of the RPM box. Evaluation of a subset of 55 patients demonstrates inter‐ and intrafraction variation over treatment. We also calculated baseline drift and duty cycle for patients and volunteers. The mean peak‐to‐peak amplitude (SD) for the patients was 1.0 (0.5) cm, and for the volunteers it was abdomen 0.8 (0.3) cm and thoracic 0.2 (0.2) cm. The mean period (SD) was 3.6 (1.0) s, 4.2 (1.1) s, and 4.1 (0.8) s, and the mean end exhale position (SD) was 60% (6), 58% (7), and 56% (7) for patient, volunteer abdomen, and volunteer thoracic, respectively. Baseline drift was greater than 0.5 cm for 40% of patients. We found statistically significant differences between the patient and volunteer groups. Peak‐to‐peak amplitude was significantly larger for patients than the volunteer abdominal measurement and the volunteer abdominal measurement is significantly larger than the volunteer thoracic measurement. The patient group also exhibited significantly larger baseline drift than the volunteer group. We also found that peak‐to‐peak amplitude was the most variable parameter for both intra‐ and interfraction motion. This database compilation can be used as a resource for expected motion when using external surrogates in radiotherapy applications.

PACS number: 87.19.Wx, 87.55.Km

## I. INTRODUCTION

Patient respiratory data is easily and readily accessible through external surrogates, which are frequently used to monitor respiratory motion. External chest wall and abdominal surrogate motion are used clinically for 4D CT,^(^ ^1^ ^,^ ^2^ ^)^ to gate images for patient setup, respiratory gated treatments, and for motion tracking.^(^ ^3^ ^,^ ^4^ ^,^ ^5^ ^,^ ^6^ ^)^

Common external surrogate systems include Real‐time Position Management and pneumatic bellows. The RPM system employs an infrared camera and small plastic box with reflective markers placed on the patient thorax or abdomen to monitor and record external motion. The bellows system consists of a deformable belt placed around the abdomen that expands and contracts with respiratory motion. The changing tension in the belt is measured to produce the respiratory signal. Both these surrogates are commercially available and currently used clinically. Ideally, internal tumor motion can be tracked with real time X‐ray imaging, but this is at the cost of increased radiation dose to patients. External surrogates can complement X‐ray‐based imaging techniques as a means to reduce the required frequency of imaging interventions.

Although external surrogates are widely used in radiotherapy applications, the extent of this motion is not well‐described in the literature. There have been few, small sample sized studies that have investigated respiratory motion parameters for external respiratory surrogates.^(^ ^7^ ^,^ ^8^ ^,^ ^9^ ^)^ I n order to fully describe this respiratory motion, a large population study is needed. To complete the picture of respiratory motion, not only do the basic extent of motion parameters, such as peak‐to‐peak amplitude, period, and end exhale phase need to be described, but also the variability of respiratory motion including inter‐ and intrafraction motion and baseline drift.

While gating is traditionally used with lung, liver, and abdominal patients, it is increasingly applied to breast cancer treatments.^(^ ^10^ ^)^ There is limited respiratory data specific to this population. The breast cancer patient population may have different respiratory qualities based on potentially better lung function, the fact that patients are often younger, and the almost exclusively female population. The inclusion of the healthy volunteer study provides a different population group from the commonly studied lung cancer patient population. Lung cancer patients often have compromised lung function and the general characteristics of their breathing patterns will not necessarily be representative of all populations.^(^ ^11^ ^)^ It has been suggested that women may breathe more with their thorax than men.^(^ ^12^ ^)^ The thoracic respiratory motion will be more important for breast cancer patients than abdominal motion that is more commonly studied. Motion of the thorax can crucially impact the heart dose in left‐sided breast cancer patients.^(^ ^13^ ^)^ Dose homogeneity in the breast has been shown to decrease with organ motion due to respiration.^(^ ^14^ ^,^ ^15^ ^)^ In the specific case of breast cancer patients, the external surrogate motion should be directly related to tumor motion; however, this is not necessarily the case for other tumor locations.

With this in mind, we have analyzed a large patient database and compiled a volunteer study of respiratory motion in order to develop a population database of respiratory information. Extent of motion information including peak‐to‐peak amplitude, period, and end exhale positions, are necessary for understanding the typical ranges to expect during radiation therapy treatment. Fluctuations of parameters during typical treatment times and between treatments days are studied in order to understand the expected intra‐ and interfraction variations. Baseline drift and duty cycle are also examined. Duty cycle is a critical part of any gating program and should be optimized for efficiency and efficacy of treatment. We also determined the amplitude/ phase correspondence for the external surrogates across typical phase bins used in 4D imaging. This analysis has implications for gated radiotherapy where imaging is phase‐based and treatment is often amplitude‐based. These analyses will be valuable for both treatment planning and commissioning of external surrogates.

## II. MATERIALS AND METHODS

We obtained respiratory data from the Real Time Position Management System (RPM, Varian Medical Systems, Palo Alto, CA). The two main components of the RPM system are a marker block and a tracking camera.^(^ ^16^ ^)^ The camera is a charge coupled device (CCD) with an infrared (IR) emitter and was installed on the ceiling of the treatment rooms and on the foot of the bed in the simulation room. Two reflective circular markers on the plastic cuboid block are tracked simultaneously to measure the calibrated vertical motion. We used this RPM set up to study two different populations: patients and volunteers.

The RPM patient database consists of traces from lung, chest, and abdominal patients (Table 1). The data were acquired with RPM block placed between the xiphoid process and the umbilicus at the position of largest respiratory motion. There are over 1000 patient traces from 120 individual patients.

**Table 1:** Patient characteristics.

	*Patients*	*Volunteers*
Mean age (range)	68.6 (4–92) years	35 (22–65)
Male/female	57/63	5/25
Lung/Chest/Other^a^	80/33/7	N/A
R/L vs C	45/53 vs. 15	N/A

a Other includes liver, abdominal, Hodgkin's, seminoma (blood vessels), and spine.

The volunteer study consisted of 30 (25 female and 5 male) healthy participants without lung cancer. We recorded RPM data at two respiratory positions: the abdomen, similar to the clinical setup, and the thorax on the sternum at the nipple line, which provides an estimate of breast motion. These two placements allow for comparison between abdominal and thoracic respiratory motion. For five of the female volunteers, we also rotated the RPM camera, block placement, and volunteer couch position 90° to assess the superior–inferior (SI) component of the motion.

Each patient and volunteer breathing trace consists of up to six minutes of respiratory data. For complete population data without weighting bias, we analyzed the single longest trace for each patient. We excluded null data traces where the IR signal was blocked or data were incorrectly recorded. We also excluded datasets with a large number of breath holds that were not representative of normal breathing, and any datasets with less than one minute of respiratory data.

The University of Calgary's Conjoint Faculties Research Ethics Board approved these studies.

### A. Population statistics

From the RPM data, we found the peak‐to‐peak amplitude, period, and end exhale phase for each subject. End exhale is the phase of the local minima for an individual breathing cycle. Analysis of variance (ANOVA) was conducted for amplitude, period, and end exhale phase for the three groups: patient abdominal, volunteer abdominal, and volunteer thoracic. Two‐tailed t‐tests determined which parameters were statistically different, with a 5% significance level. We performed all analyses in MATLAB (The MathWorks, Natick, MA).

**Figure 1:**
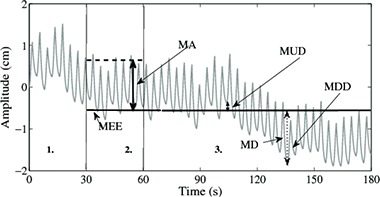
The first 30 seconds of data (a) are discarded to allow subjects to relax into restful breathing and to exclude any set up changes such as couch shifts and patient adjustments; (b) for the next 30–60 s, the mean location of end exhale (MEE) and cycle amplitude (MA) were found; the maximum upward (MUD) (c) and downward drift (MDD) were calculate from MEE and the maximum drift (MD) and percent drift determined.

Baseline drift is defined as the change in the vertical position of the local minima (end exhale) of the respiratory cycle^(^ ^2^ ^)^ and was calculated for each trace, as shown in Fig. 1. Dependency of baseline drift on tumor location was examined by comparing both the absolute and percent baseline drift between right/left and centrally located tumors. Duty cycle is an important measure of treatment efficiency in gated radiotherapy. It is defined as the ratio of beam‐on time to treatment delivery time. In this study, we calculated duty cycle of amplitude‐based gating,^(^ ^17^ ^)^ for gating windows of 10%–60%.

In order to investigate amplitude variation across common phase bins employed by applications such as 4D CT, we examined the spread of amplitude points at typical phase windows. Each patient trace was separated into phase‐binned cycles (phase 1–101) and then these were split into phase bins of 1–11, 11–21,…, and 91–101. The variation across each bin is displayed using box and whisker plots.

### B. Patient statistics over time

To investigate inter‐ and intrafraction motion, we examined patient respiratory traces from three different treatment days, each spaced at least one week and up to two weeks apart. Fifty‐five of 125 patients matched these criteria. We evaluated the inter‐ and intrafraction variability for period, peak‐to‐peak amplitude, and end exhale phase. The interfraction variation was defined as the standard deviation (SD) of all data over the three days of treatment, and the intrafraction variation as the SD from each treatment day. We evaluated the mean change over time by averaging the three daily intrafraction measurements. The coefficient of variation ((CV=SD/mean*100%) provides a standardized comparison between the three parameters: period, peak‐to‐peak amplitude, and end exhale phase. The correlation coefficient was calculated between the CV of peak‐to‐peak amplitude, period, and end exhale phase to determine if variability of one parameter had a definitive impact on another. Baseline drift was also analyzed over time for two subsets of the population: 55 patients over three different treatment days and 10 patients over five different treatment days.

## III. RESULTS

### A. Population statistics

**Figure 2:**
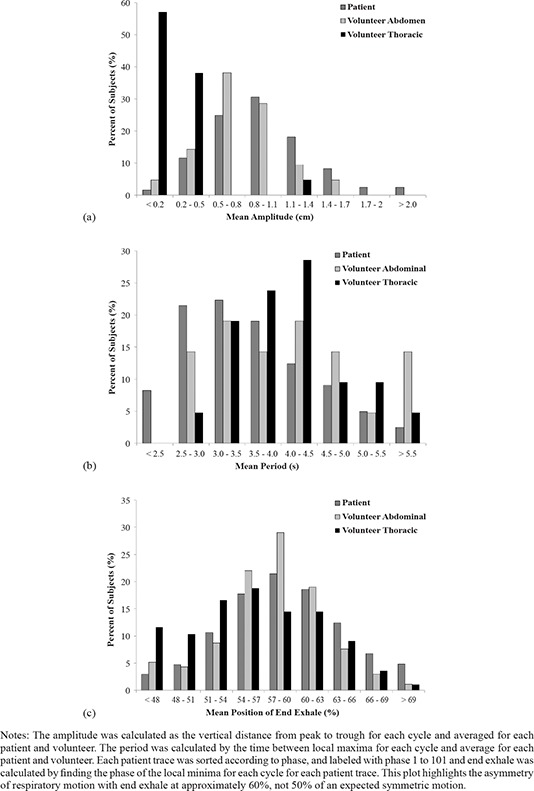
Notes: The amplitude was calculated as the vertical distance from peak to trough for each cycle and averaged for each patient and volunteer. The period was calculated by the time between local maxima for each cycle and average for each patient and volunteer. Each patient trace was sorted according to phase, and labeled with phase 1 to 101 and end exhale was calculated by finding the phase of the local minima for each cycle for each patient trace. This plot highlights the asymmetry of respiratory motion with end exhale at approximately 60%, not 50% of an expected symmetric motion. Histograms of peak‐to‐peak amplitude (cm), period (s), and end exhale phase (%) are shown for patients (dark grey), volunteers abdominal measurement (light grey), and volunteer thoracic measurement (black).

The population mean and standard deviation of the peak‐to‐peak amplitude, period, and end exhale phase are shown in Table 2, and the histograms comparing these measurements for patients, volunteer abdominal, and volunteer thoracic are shown in Figs. 2(a), 2(b), and 2(c). Peak‐to‐peak amplitude, period, and end exhale all have similar, approximately normal, distributions. Only the female volunteers were included in the analysis, because for the males in our study, the motion of the RPM box placed on the thorax was not reliably detectable. For the volunteer study, the superior–inferior (SI) motion of a small subset of the volunteers was analyzed. The mean peak‐to‐peak SI amplitude of these five volunteers was 0.1 cm (range of 0–0.2 cm), the mean period was 3.6 s (2.6–4.8 s), and the mean end exhale phase was 56.7% (52.9%–61.3%). The SI motion was smaller in each volunteer than the corresponding measurement of either the thoracic or abdominal motions in the anterior–posterior direction.

**Table 2:** Mean, median, and standard deviation (SD) of peak‐to‐peak amplitude, period, and end exhale phase for patients and volunteers, both abdominal and thoracic placement.

		*Patient*	*Volunteer Abdomen*	*Volunteer Thoracic*
Amplitude (cm)	Mean	1.0	0.8	0.2
	Median	0.9	0.8	0.2
	SD	0.5	0.3	0.2
Period (s)	Mean	3.6	4.2	4.1
	Median	3.5	4.0	4.1
	SD	1.0	1.1	0.8
End exhale (%)	Mean	59.5	57.7	56.1
	Median	59.0	58.0	56.0
	SD	6.3	6.9	6.8

The differences between the abdominal motion of the volunteers and patients, and the thoracic and abdominal motion of the volunteers were tested for statistical significance. The period was not statistically different between these groups. Patients had significantly greater abdominal peak‐to‐peak amplitude (p=0.04) than volunteers' abdominal motion. The volunteers' thoracic motion was significantly smaller than their abdominal motion (p=0.01). Statistically, the end exhale phase for patients occurred later in the breathing cycle than for volunteers (p<0.001). A systematic phase difference in volunteers' thoracic and abdominal end exhale phase was observed, with the abdominal end exhale position at a later phase than the thoracic position (p<0.001).

**Figure 3:**
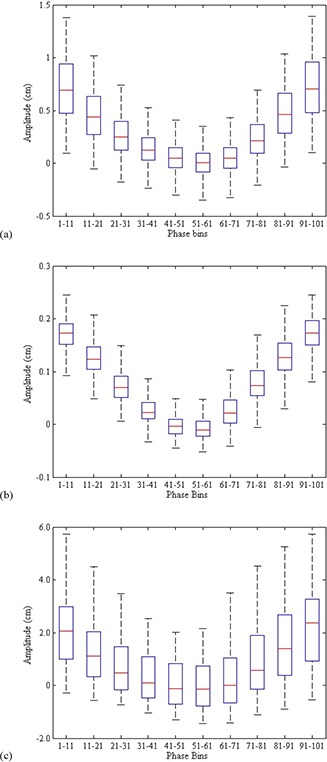
The amplitude/phase correspondence is shown for all patients (a), as well as the patient with the minimal variation (b) and the maximal variation (c).

The amplitude/phase correspondence across typical imaging phase bins was analyzed. Figure 3(a) shows box and whisker plots for each of the phase bins for the amplitudes of all patients. The box portion of the plot represents the 25th and 75th quartiles and the central line the 50th quartile (median). The whisker portion is the 95% spread of the data. The spread of the exhale bins is smaller than the inhale bins, which is consistent with findings showing end exhale as the more stable position. The last two graphs in Fig. 3 show the variation found from single patients. Figure 3(b) shows the patient with the minimal standard deviation of amplitude in each bin, while Fig. 3(c) shows the patient with maximal variation. Figure 3(c) represents a patient that falls beyond the 95% of the data shown in Fig. 3(a).

**Figure 4:**
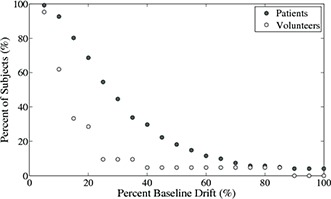
Cumulative plot of percent baseline drift of patients (black circles) and volunteers (light grey circles) is shown. The percent baseline is calculated as shown in Fig 1.

Figure 4 shows the baseline drift for patient and volunteer abdominal measurements. The shapes of the curves are very similar; however, patient baseline drift is larger than that of the volunteers (p=0.008). The absolute baseline drift was measured up to 2 cm in the patient group and 0.3 cm in volunteer group. Over 40% of patients show baseline drifts of 30% of the amplitude, while 10% of patients show percent baseline drifts greater than 60% of the amplitude. The absolute baseline was greater than 0.5 cm for 40% of patients. The percent baseline drift of the volunteers is much smaller. Only 10% of volunteers have a baseline drift of 30% and less than 5% have a baseline drift greater than 60%.

A subanalysis compared the correlation of baseline drift to tumor position for the chest/ lung cancer patients between centrally and right/left located tumors. We found no statistically significant differences between either absolute baseline drift (central: 19±16mm and right/left: 32±31mm) or percent baseline drift (central: 20.9%±24.5% and right/left: 50.2%±80.0%). Baseline drift is hypothesized to be associated with stress^(^ ^2^ ^)^ and the nonstatistically significant difference between central and peripheral tumors is consistent with hypothesis, as the stress levels of the patients would not necessarily be different across those two groups.

**Figure 5:**
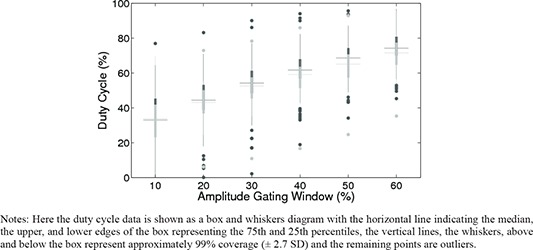
Notes: Here the duty cycle data is shown as a box and whiskers diagram with the horizontal line indicating the median, the upper, and lower edges of the box representing the 75th and 25th percentiles, the vertical lines, the whiskers, above and below the box represent approximately 99% coverage (±2.7 SD) and the remaining points are outliers. Duty cycle of patients (dark grey) and volunteers (light grey).

The duty cycles for both the volunteers (abdominal) and the patients are shown in Fig. 5 and the results are similar. The duty cycle spread is greater in the patient population. For the volunteers, the outliers represent the same two volunteers: one at the upper bound and one at the lower.

### B. Patient statistics over time

**Figure 6:**
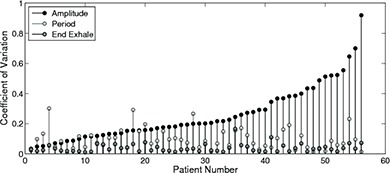
Interfraction coefficient of variation is shown for amplitude (black), period (light grey), and end exhale (dark grey). The data are presented in order of increasing amplitude variability. For the coefficient of variation, the standard deviation was calculated from three different treatment days, each a week apart. There is no correlation between the coefficient of variation for amplitude, period, and end exhale.

**Figure 7:**
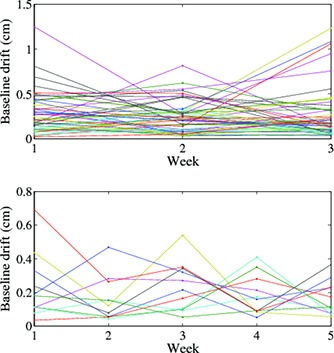
Baseline drift for: (a) 50 patients with three measurements each a week apart, and (b) 10 patients, five measurements each a week apart. There is no obvious trend of increasing or decreasing baseline drift over the course of treatment.

We calculated both inter‐ and intrafraction variability for a subset of 55 patients. Table 3 gives the mean, standard deviation, and coefficient of variation (CV) for inter‐ and intrafraction motion. The CV allows for comparison between the three parameters and indicates that end exhale is more stable between and within fractions than amplitude or period. The CV is larger for interfraction motion than intrafraction motion. Figure 6 shows that the variability of one parameter is not correlated with the variability of the others with correlation coefficients of 0.10 (end exhale and period), 0.08 (amplitude and period), and 0.12 (end exhale and amplitude). Figures 7(a) and 7(b) show the baseline drift for the 50 patients over three days and the 10 patients over five days. Baseline drift is found to fluctuate over time, with no clear increasing or decreasing trends.

**Table 3:** Intra‐ and interfraction variability of peak‐to‐peak amplitude, period, and end exhale for 55 patients.

*Intrafraction Motion*	*Amplitude (cm)*	*Period (s)*	*End Exhale (%)*
Mean	0.9	3.6	60.3
SD	0.2	0.4	2.3
CV (%)	22.5	10.2	3.8
*Interfraction Motion*			
Mean	0.9	3.6	60.3
SD	0.3	0.9	4.9
CV (%)	37.0	24.3	8.1

## IV. DISCUSSION

Our study examines the external motion of the chest wall and not internal tumor motion. This data is one‐dimensional and therefore cannot account for hysteresis, but should portray similar characteristics to the internal motion. External surrogates are beneficial because the data are easily accessible; however, caution must be taken to verify the internal–external correlation prior to clinical applications. Our measurements and analysis provide accumulation respiratory motion data that can be consulted for typical ranges of motion and variability.

Respiratory motion measures, including peak‐to‐peak amplitude and period, have been explored in the literature for external surrogates with smaller patient numbers.^(^ ^7^ ^,^ ^8^ ^,^ ^9^ ^,^ ^18^ ^)^ Our results for mean (SD) for amplitude, 1.0 (0.5) cm, and period, 3.7 (1.0) s, fall into the range of motion found for external motion range of amplitudes of 3 mm to 3 cm^(^ ^8^ ^,^ ^9^ ^)^ and a range of period of 4.8 (1.2) s.^(^ ^18^ ^)^

Only one paper, to the best of our knowledge, shows a comparison between lung cancer patients and volunteers. Cai et al.^(^ ^19^ ^)^ investigated internal lung motion in seven healthy volunteers and five lung cancer patients. The differences between the two groups were not explicitly examined. Data of the two groups were combined into a single analysis that provided no comparison between lung cancer patients and healthy volunteers. Using the data provided in their manuscript, we calculated the mean amplitude of 0.90±0.29 cm and 0.90±0.30 cm for volunteers and patients, respectively. Although, their analysis was performed on an internal dataset, the relationship between the magnitude volunteer and patient respiratory motion is consistent with our results of 0.8±0.2 cm and 1.0±0.5 cm for volunteers and patients.

The use of an external surrogate is potentially closer to reality for the early stage breast patient population because the trajectory of the tumor will be more true to the trajectory of the external surrogate. Our volunteer group represents this population in such characteristics as age, lung function, and activity level ranges. The thoracic position of respiratory motion measured for the volunteers is potentially representative of breast motion, but does not account for the difference in stress levels that a breast cancer patient may experience compared to a volunteer. Chopra et al.^(^ ^20^ ^)^ found, for a study of five breast cancer patients, the maximum thoracic amplitude during normal breathing to be approximately 0.2 cm and during deep breathing to be up to 0.5 cm. This result is consistent with the amplitude of 0.3±0.2 cm from our volunteer study.

Our results show that the end exhale position of both the volunteers and patients was close to 60% phase. For more than 95% of subjects, the majority of the respiratory cycle is spent in exhale. This indicates the asymmetry between time spent in exhale and inhale during the respiratory cycle. The assumption that end exhale occurs at 50% phase^(^ ^21^ ^,^ ^22^ ^)^ is often used, but this simplification ignores the asymmetric quality of respiratory motion. Lujan et al.^(^ ^23^ ^)^ explored the impact of asymmetry of motion for 10 liver patients by showing that the effective volume change of the uninvolved liver can vary between ‐5% and 10% for an asymmetric motion with 70% end exhale phase, while smaller variations are seen when the motion is more symmetric. Lujan and colleagues concluded that there was a small dosimetric impact on conventional radiation therapy, but a greater impact could be seen for highly conformal treatments such as IMRT.

The end exhale phase differed with statistical significance between all three groups. Between patients and volunteers abdominal measurement the average difference is small (<1%) so there is little clinical significance in this finding. In the volunteer group, a statistically significant difference in end exhale phase was found between abdominal and thoracic breathing (2%). The difference between thoracic and abdominal phase for volunteers could suggest a phase shift between internal/external correlation. Again this average difference is quite small, so the clinical impact is minimal. The maximal difference of one volunteer was over 12% and could have a clinical significance if the thoracic motion was used as a surrogate for abdominal motion (or vice versa).

The results for interfraction motion show significant changes in amplitude and period between treatment fractions. Amplitude had the largest interfraction variation with a standard deviation of 5 mm. Korreman et al.^(^ ^24^ ^)^ looked at interfraction variability for 17 patients and found similar results with 1.6 to 8.1 mm standard deviations over the entire course of treatment for external respiratory amplitude. We found that on average, the interfraction variability was larger than the intrafraction variability. This is consistent with results for end exhale phase by Juhler‐Nottrup et al.^(^ ^25^ ^)^ that showed the interfraction variation was significantly larger than the intrafraction variation.

Lujan et al.^(^ ^23^ ^)^ looked at the clinical impact of intrafraction variability of amplitude and found that small changes (< 3 mm) in amplitude may not result in clinically significant changes, but larger variations (> 5 mm) can lead to significant changes. Our average intrafraction standard deviation is 3 mm, with 12/55 patients having standard deviation greater than 5 mm. This indicates approximately 20% of our patients may see clinically significant changes due to their intrafraction variability.

Baseline drift requires monitoring during both gated and non‐gated treatments because of the potential for a geometric miss. It is important during commissioning to ensure that proper systems are in place to handle baseline drifts. When drift occurs during respiratory gating, the treatment is stopped and the gating window is readjusted, increasing uncertainty in the treatment and overall delivery time.^(^ ^26^ ^)^ We found a statistically significant difference in baseline drift between patients and volunteers. This indicates that baseline drift could be correlated to lung function and, if this is the case, patients with poor lung function should be closely monitored if using gating or other complex radiotherapy methods with small internal target volume (ITV) margins. The difference in baseline drift between the two populations could also be due to disparity of stress/relaxation levels between patients with the stress of cancer treatment and volunteers participating in a lower stress study. Rietzel et al.^(^ ^2^ ^)^ explored the cause of baseline drift and found that an important cause could be relaxation of the patient during the procedure or changes between abdominal and thoracic breathing, while also concluding that the influence on internal motion is unclear. Baseline drift has been demonstrated in internal tumor motion studies^(^ ^27^ ^,^ ^28^ ^)^ and significant shifts are found in some patients.^(^ ^5^ ^,^ ^28^ ^)^ A definitive correlation between external and internal drift is not available and must be investigated on a patient‐by‐patient basis.^(^ ^11^ ^,^ ^29^ ^)^

We calculated duty cycle for the patients and volunteers from the position of end exhale for simulated amplitude‐based gating. Most clinical gating studies use a 25%–50% duty cycle.^(^ ^21^ ^)^ We found that this corresponded to a median amplitude gating window of 10%–30% for both patients and volunteers. On average, this gating window is very small, approximately 1–3 mm of external motion, requiring external surrogate monitoring with submillimeter accuracy.

## V. CONCLUSIONS

We analyzed external chest wall respiratory motion for 120 patient and 25 volunteer traces to determine mean values for peak‐to‐peak amplitude, period, and end exhale phase. Statistically and potentially clinically significant differences were found for both peak‐to‐peak amplitude between the patient and volunteer abdominal measurements (1.0±0.5 cm vs. 0.8±0.3 cm), and between the volunteer abdominal and thoracic measurements (0.8±0.3 cm vs. 0.2±0.2 cm). As well, inter‐ and intrafraction variability was evaluated for 55 patients and it was found that variability between fractions was larger than variability within a fraction, and that amplitude was more variable than period and end exhale phase for both inter‐ and intrafraction measurements. This study provides a database of parameters to use when testing implementation of techniques that rely on external surrogates. It also provides a useful comparison between patients with mainly liver, lung, and abdominal cancer and a volunteer population that could more accurately represent breast cancer patients.

## ACKNOWLEDGMENTS

The authors thank Ginelle Johnston for her valuable assistance on this project.

## References

[1] Vedam SS , Keall PJ , Kini VR , Mostafavi H , Shukla HP , Mohan R . Acquiring a four‐dimensional computed tomography dataset using an external respiratory signal. Phys Med Biol. 2003;48(1):45–62. 12564500 10.1088/0031-9155/48/1/304

[2] Rietzel E , Pan T , Chen GT . Four‐dimensional computed tomography: image formation and clinical protocol. Med Phys. 2005;32(4):874–89. 15895570 10.1118/1.1869852

[3] Berbeco RI , Nishioka S , Shirato H , Chen GT , Jiang SB . Residual motion of lung tumours in gated radiotherapy with external respiratory surrogates. Phys Med Biol. 2005;50(16):3655–67. 16077219 10.1088/0031-9155/50/16/001

[4] Schweikard A , Shiomi H , Adler J . Respiration tracking in radiosurgery. Med Phys. 2004;31(10):2738–41. 15543778 10.1118/1.1774132

[5] Korreman S , Mostafavi H , Le QT , Boyer A . Comparison of respiratory surrogates for gated lung radiotherapy without internal fiducials. Acta Oncol. 2006;45(7):935–42. 16982560 10.1080/02841860600917161

[6] Cho B , Suh Y , Dieterich S , Keall PJ . A monoscopic method for real‐time tumour tracking using combined occasional x‐ray imaging and continuous respiratory monitoring. Phys Med Biol. 2008;53(11):2837–55. 18460750 10.1088/0031-9155/53/11/006

[7] George R , Vedam SS , Chung TD , Ramakrishnan V , Keall PJ . The application of the sinusoidal model to lung cancer patient respiratory motion. Med Phys. 2005;32(9):2850–61. 16266099 10.1118/1.2001220

[8] Fuji H , Asada Y , Numano M , et al. Residual motion and duty time in respiratory gating radiotherapy using individualized or population based windows. Int J Radiat Oncol Biol Phys. 2009;75(2):564–70. 19735882 10.1016/j.ijrobp.2009.03.074

[9] Nishioka S , Nishioka T , Kawahara M , et al. Exhale fluctuation in respiratory‐gated radiotherapy of the lung: a pitfall of respiratory gating shown in a synchronized internal/external marker recording study. Radiother Oncol. 2008;86(1):69–76. 18077028 10.1016/j.radonc.2007.11.014

[10] Korreman SS , Pedersen AN , Nottrup TJ , Specht L , Nystrom H . Breathing adapted radiotherapy for breast cancer: comparison of free breathing gating with the breath‐hold technique. Radiother Oncol. 2005;76(3):311–18. 16153728 10.1016/j.radonc.2005.07.009

[11] Ozhasoglu C and Murphy MJ . Issues in respiratory motion compensation during external‐beam radiotherapy. Int J Radiat Oncol Biol Phys. 2002;52(5):1389–99. 11955754 10.1016/s0360-3016(01)02789-4

[12] Wang CS and Josenhans WT . Contribution of diaphragmatic‐abdominal displacement to ventilation in supine man. J Appl Physiol. 1971;31(4):576–80. 5111005 10.1152/jappl.1971.31.4.576

[13] Remouchamps V , Vicini F , Sharpe M , Kestin L , Martinez A , Wong J . Significant reductions in heart and lung doses using deep inspiration breath hold with active breathing control and intensity‐modulated radiation therapy for patients treated with locoregional breast irradiation. Int J Radiat Oncol Biol Phys. 2003;55(2):392–406. 12527053 10.1016/s0360-3016(02)04143-3

[14] Menon G , Pudney D , Smith W . Dosimetric evaluation of breast radiotherapy in a dynamic phantom. Phys Med Biol. 2011;56(23):7405–18. 22056856 10.1088/0031-9155/56/23/005

[15] George R , Keall PJ , Kini VR , et al. Quantifying the effect of intrafraction motion during breast IMRT planning and dose deliver. Med Phys. 2003;30(4):552–62. 12722807 10.1118/1.1543151

[16] Berson A , Emery R , Rodriguez L , et al. Clinical experience using respiratory gated radiation therapy: comparison of free‐breathing and breath‐hold techniques. Int J Radiat Oncol Biol Phys. 2004;60(2):419–26. 15380575 10.1016/j.ijrobp.2004.03.037

[17] Coolens C , Webb S , Shirato H , Nishioka K , Evans PM . A margin model to account for respiration‐induced tumour motion and its variability. Phys Med Biol. 2008;53(16):4317–30. 18653921 10.1088/0031-9155/53/16/007

[18] Otani Y , Fukuda I , Tsukamoto N , et al. A comparison of the respiratory signals acquired by different respiratory monitoring systems used in respiratory gated radiotherapy. Med Phys. 2010;37(12):6178–86. 21302774 10.1118/1.3512798

[19] Cai J , McLawhorn R , Read PW , et al. Effects of breathing variation on gating window internal target volume in respiratory gated radiation therapy. Med Phys. 2010;37(8):3927–34. 20879555 10.1118/1.3457329

[20] Chopra S , Dinshaw KA , Kramble R , Sarin R . Breast movement during normal and deep breathing, respiratory training and set up errors: implications for external beam partial breast irradiation. Br J Radiol. 2006;79(945):766–73. 16940376 10.1259/bjr/98024704

[21] Vedam SS , Archambault L , Starkschall G , Mohan R , Beddar S . Determination of prospective displacement‐based gate threshold for respiratory‐gated radiation delivery from retrospective phase‐based gate threshold selected at 4D CT simulation. Med Phys. 2007;34(11):4247–55. 18072489 10.1118/1.2794169

[22] Santoro JP , Yorke E , Goodman KA , Mageras GS . From phase‐based to displacement‐based gating: a software tool to facilitate respiration‐gated radiation treatment. J Appl Clin Med Phys. 2010;10(4):2982–97. Retrieved May 30, 2011 from http://www.jacmp.org 10.1120/jacmp.v10i4.2982 PMC2826245 19918227

[23] Lujan AE , Balter JM , TenHaken RK . A method for incorporating organ motion due to breathing into 3D dose calculations: sensitivity to variations in motion. Med Phys. 2003;30(10):2643–49. 14596301 10.1118/1.1609057

[24] Korreman SS , Juhler‐Nottrup T , Boyer A . Respiratory gated beam delivery cannot facilitate margin reduction, unless combined with respiratory correlated image guidance. Radiother Oncol. 2008;86(1):61–68. 18039549 10.1016/j.radonc.2007.10.038

[25] Juhler‐Nottrup T , Korreman SS , Pedersen AN , et al. Intra‐ and interfraction breathing variation during curative radiotherapy for lung cancer. Radiother Oncol. 2007;84(1):40–48. 17588697 10.1016/j.radonc.2007.05.026

[26] Aristophanous M , Rottmann J , Park SJ , Nishioka S , Shirato H , Berbeco RI . Image‐guided adaptive gating of lung cancer radiotherapy: a computer simulation study. Phys Med Biol. 2010;55(15):4321–33. 20647609 10.1088/0031-9155/55/15/009

[27] Shirato H , Suzuki K , Sharp G , et al. Speed and amplitude of lung tumor motion precisely detected in four‐dimensional setup and in real‐time tumor tracking radiotherapy. Int J Radiat Oncol Biol Phys. 2006;64(4):1229–36. 16504762 10.1016/j.ijrobp.2005.11.016

[28] Seppenwoolde T , Berbeco R , Nishioka S , Shirato H , Heijmen B . Accuracy of tumor motion compensation algorithm from a robotic respiratory tracking system: a simulation study. Med Phys. 2007;34(7):2774–84. 17821984 10.1118/1.2739811

[29] Keall PJ , Mageras GS , Balter JM , et al. The management of respiratory motion in radiation oncology report of AAPM Task Group 76. Med Phys. 2006;33(10):3874–900. 17089851 10.1118/1.2349696

